# Etiologies and Resistance Profiles of Bacterial Community-Acquired Pneumonia in Cambodian and Neighboring Countries’ Health Care Settings: A Systematic Review (1995 to 2012)

**DOI:** 10.1371/journal.pone.0089637

**Published:** 2014-03-13

**Authors:** Sophie Goyet, Erika Vlieghe, Varun Kumar, Steven Newell, Catrin E. Moore, Rachel Bousfield, Heng C. Leang, Sokheng Chuop, Phe Thong, Blandine Rammaert, Sopheak Hem, Johan van Griensven, Agus Rachmat, Thomas Fassier, Kruy Lim, Arnaud Tarantola

**Affiliations:** 1 Epidemiology unit, Institut Pasteur du Cambodge, Phnom Penh, Cambodia; 2 Institute of Tropical Medicine, Antwerp, Belgium; 3 Angkor Hospital for Children, Siem Reap, Cambodia; 4 Naval Medical Research Unit2, Phnom Penh, Cambodia; 5 Wellcome Trust Major Overseas Programme, Mahidol-Oxford Tropical Medicine Research Unit, Bangkok, Thailand; 6 Centre for Clinical Vaccinology and Tropical Medicine, Churchill Hospital, Oxford University, Oxford, United Kingdom; 7 National Institute of Public Health, Phnom Penh, Cambodia; 8 Sihanouk Hospital Center of HOPE, Phnom Penh, Cambodia; 9 Hopital Necker-Enfants malades service des Maladies Infectieuses et Tropicales, APHP, Paris, France; 10 University of Health Sciences, Faculty of Medicine, Phnom Penh, Cambodia; University of Padova, Medical School, Italy

## Abstract

**Objectives:**

Community-acquired pneumonia (CAP) is one of the most important causes of morbidity and mortality worldwide. Etiological data for Cambodia is scarce. We aimed to describe the main etiological agents causing CAP, and their resistance patterns in Cambodia and the greater Mekong region.

**Methods:**

A review of bacterial etiologies of CAP and antimicrobial resistance in Cambodia and neighboring countries was conducted via: (1) a systematic review of published literature in all NCBI databases using Pubmed, Google scholar, EMBASE, the World Health Organization and the Cambodian Ministry of Health libraries; (2) a review of unpublished data from Cambodia provided by national and international stakeholders working at different tiers of the healthcare system.

**Results:**

Twenty three articles and five data sources reported etiologies for 5919 CAP patients diagnosed between May 1995 and December 2012, including 1421 (24.0%), 3571 (60.3%) and 927 (15.7%) from Cambodia, Thailand and Vietnam, respectively. *Streptococcus pneumoniae* and *Haemophilus influenzae* were the most common pathogens ranking among the five most prevalent in 12 and 10 studies, respectively. Gram-negative bacteria such as *Burkholderia pseudomallei* and *Klebsiella pneumoniae* were also frequently diagnosed, particularly in bacteremic CAP in Thai adults and Cambodian children. In Thailand and Vietnam, *Mycoplasma pneumoniae* and *Chlamydia pneumoniae* were frequently identified in settings using indirect laboratory testing.

**Conclusions:**

Based on this analysis, CAP data in Cambodia seems to present etiological and resistance profiles comparable to those of neighboring countries. Findings have been shared with the national authorities upon the revision of the national therapeutic guidelines and were disseminated using a specially created website.

## Introduction

Despite a substantial reduction since 1990, community-acquired pneumonia (CAP) and other lower respiratory infections (LRI) still rank as the second most frequent cause of all-age premature deaths at the global level [1]. CAP and other LRIs remain a leading cause of morbidity among children <5 years, causing 12 million episodes of hospital admissions a year [2], and 1.6 million deaths in young children [3] worldwide. CAP also imposes a substantial disease burden on older children [4] and adults [5], [6]. Improving the therapeutic management of pneumonia has been defined by international experts as one of the top priorities to decrease the CAP burden and improve health worldwide [7].

CAP can be difficult to diagnose clinically [8] and to characterize radiologically, in particular in patients with underlying pulmonary conditions. Etiological diagnosis may be influenced by available microbiological testing methods or impeded by antibiotic intake prior to testing [9]. Assessing severity can also be challenging [10]. Finally, in a developing country setting, further obstacles relate to the timely access of well-equipped facilities, with affordable services, including laboratory and radiology, and staffed with well-trained health teams.

In Cambodia, a low-income Southeast Asian country of about 14 million inhabitants with an annual gross national income per capita of 880 USD [11], the LRI attack rate in Cambodian children <5 years was estimated at 6% over a 2-week period in 2010 [12]. According to a national survey, Cambodian mothers deem that ‘respiratory diseases’ are the third leading cause of death in young children [13]. The most recent projection estimated that in 2008, nearly 10 000 of the 22 000 deaths among children ≤5 years were due to CAP [3]. Repeated Cambodian demographic health surveys have not explored the LRI burden in older children or adults [12], [13]. Passive surveillance data on acute LRI within Cambodia are published in a national monthly bulletin but may be biased toward upper respiratory tract infections and viral infections [14]. Moreover, many studies describe the burden of tuberculosis (TB) in Cambodia [15], [16], but little is known of bacterial etiologies of non-TB pneumonia. Several non-governmental organizations and hospitals in Cambodia routinely collect clinical data but CAP is not routinely microbiologically-confirmed as diagnostic laboratories with culture facilities are very scarce in the country. Research organizations have been conducting studies on specific research questions, but there are no structures in place yet for routinely sharing of data or experiences. Up to 2012, Cambodian physicians were treating CAP using empirical treatment guidelines developed in 1999, without taking into account bacterial resistance emergence [17], [18]. However, in 2012, the Ministry of Health started a revision process of these national guidelines for CAP.

With the aim to provide Cambodian health policymakers with contextual and updated evidence-based information on CAP, a group of national and international clinicians and epidemiologists in Cambodia convened under the auspices of Institut Pasteur du Cambodge (IPC). This group -called the Community-Acquired Lung Infections, Bacteria and Antimicrobial Network, (CALIBAN) conducted a systematic review of published and unpublished data in Cambodia and neighboring countries on: (1) bacterial etiologies of non-TB CAP; and (2) the antimicrobial resistance patterns of the most prevalent pathogens causing CAP.

## Methods

### 1. Published (“White”) Literature Review

In May 2012, all National Center for Biotechnology Information’s (NCBI) databases were searched using Pubmed, with no restriction dates or languages and following the search strategy reported in Document S1. We also searched the Google scholar database (restricting the search on papers published since 2000), the World Health Organization and the Cambodian Ministry of Health (MOH) online libraries [19], [20], [21]. Finally, we searched the references’ list of eligible papers and directly contacted their authors. The EMBASE database was also searched in January 2014, to complete the review, using the key words ‘community-acquired pneumonia’(drug resistance, epidemiology and etiology)’, ‘Cambodia’, ‘Laos’, ‘Vietnam’, and ‘Laos’ looking for articles published until January 2013.

We excluded animal health studies, studies performed beyond the targeted geographical area, case reports and studies not focusing on pneumonia. We also excluded references on hospital-acquired infections, on pulmonary TB or CAP in immuno-compromised patients. Finally, we excluded studies presenting only virologic, immunologic or genetic results on pneumonia and studies conducted prior to 1990. Two epidemiologists independently selected the articles (SG and AT), with no disagreement. Data were extracted in duplicate using a pre-defined data sheet.

### 2. Unpublished (“Grey”) Literature

**Figure 1:**
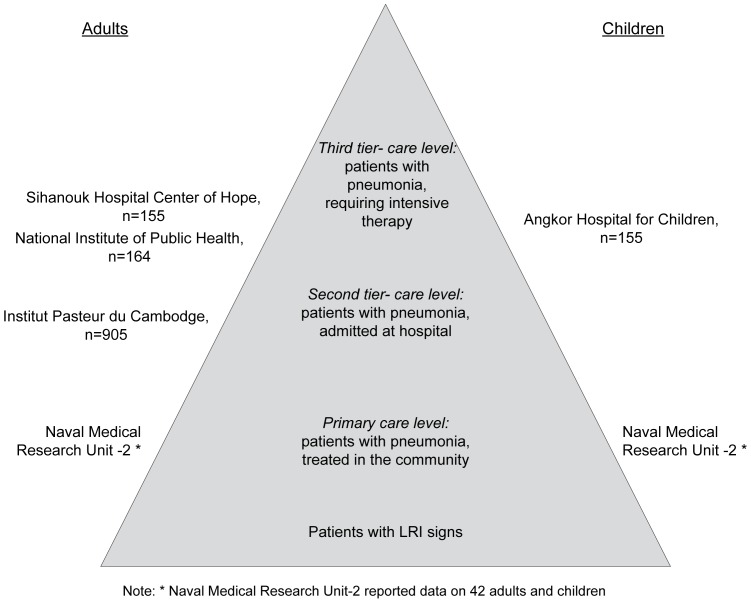
Unpublished Cambodian data sources. The Cambodian data sources were mapped within the pneumonia “iceberg”, adapted from Macfarlane [79].

Data on CAP in Cambodia were collected in 2012 through the CALIBAN group. Five CALIBAN stakeholders working at different levels of care agreed to share their “raw” data (Figure 1). The primary care level is defined as care given in the community, in health posts and health centers; the secondary care level refers to 2^nd^-tier general or provincial hospitals, while the 3^rd^ tier includes referral hospitals offering medical care to the most serious cases.

- Angkor Hospital for Children (AHC) is a referral pediatric hospital situated in Siem Reap Province. Patients aged <16 years are treated free of charge. Patients have access to laboratory and radiological investigations and results are kept electronically in the hospital database and in each patient’s individual medical record [22]. The hospital provides outpatient care to 400–500 patients/day, with approximately 1500 patients seen in the emergency room per month and 350 patients admitted per month, including 65 in the intensive care unit.
- Institut Pasteur du Cambodge (IPC) conducted a study of CAP in all-age patients, from April 2007 to July 2010 in two second-tier general hospitals [23], [24]. Bacterial etiologies were explored among ≥5 year-old patients, excluding those with known TB and positive HIV serology. Diagnosis of CAP was determined by expert pulmonologists from reviews of medical charts and chest X-rays.
- Naval Medical Research Unit-2 (NAMRU2) has been conducting sentinel surveillance of febrile illnesses in primary health care facilities of five provinces since December 2006 [25]. It is a community-based study in children and adults.
- The National Institute of Public Health (NIPH) is supported by the US Centers for Disease Control and prevention (CDC) and prospectively collects data from severe LRI patients of all ages admitted to third-tier national hospitals: two are located in Phnom Penh, and one in the nearby province of Kandal.
- Sihanouk Hospital Center of Hope (SHCH) is a 35-bed referral NGO hospital in central Phnom Penh providing free care for the poor [26]. It provides 120 000 patients with outpatient care and admits 1500 patients annually. In 2007, a prospective blood stream isolates’ study was initiated in patients presenting with Systemic Inflammatory Response Syndrome. A proportion of these patients presented clinical signs and symptoms of LRI.

Other CALIBAN stakeholders, including two partners who provide pediatric care in Cambodia were unable to contribute data.

### 3. Analysis and Assessment of Risk of Bias and Data Analysis

Descriptive statistics were computed using Stata 12 software (Stata Corp., College Station, TX, USA). Antimicrobial resistance data is reported as determined by the studies’ authors and the tests and interpretative breakpoints they used. Results are presented as numeric tables presenting frequencies and narrative synthesis.

Two independent reviewers (SG and AT) assessed the methods used by the various studies to document bacterial causes of CAP and possible bias, adapting an algorithm published by Gentile et al. [27], derived from the STROBE (Strengthening the Reporting of Observational studies in Epidemiology) checklist of essential items [28]. The case definitions for each study are presented in Table S1. Methods for selecting the study participants, the methods for measuring the bacterial pathogens’ prevalence and methods to control confounding factors were graded from low to high risk of bias (Table S2). Disagreements were resolved by consensus.

A PRISMA checklist reporting the completeness of this review is available in Checklist S1.

### 4. Ethics Statement

Ethical approvals were granted for the different sub-studies which provided unpublished data by their review boards and/or the Cambodian National Ethics Committee. The NAMRU study has been conducted in compliance with all applicable federal regulations governing the protection of human subjects in research (Protocol NAMRU2.2005.0004).

## Results

### 1. “White Data” Inclusions

**Figure 2:**
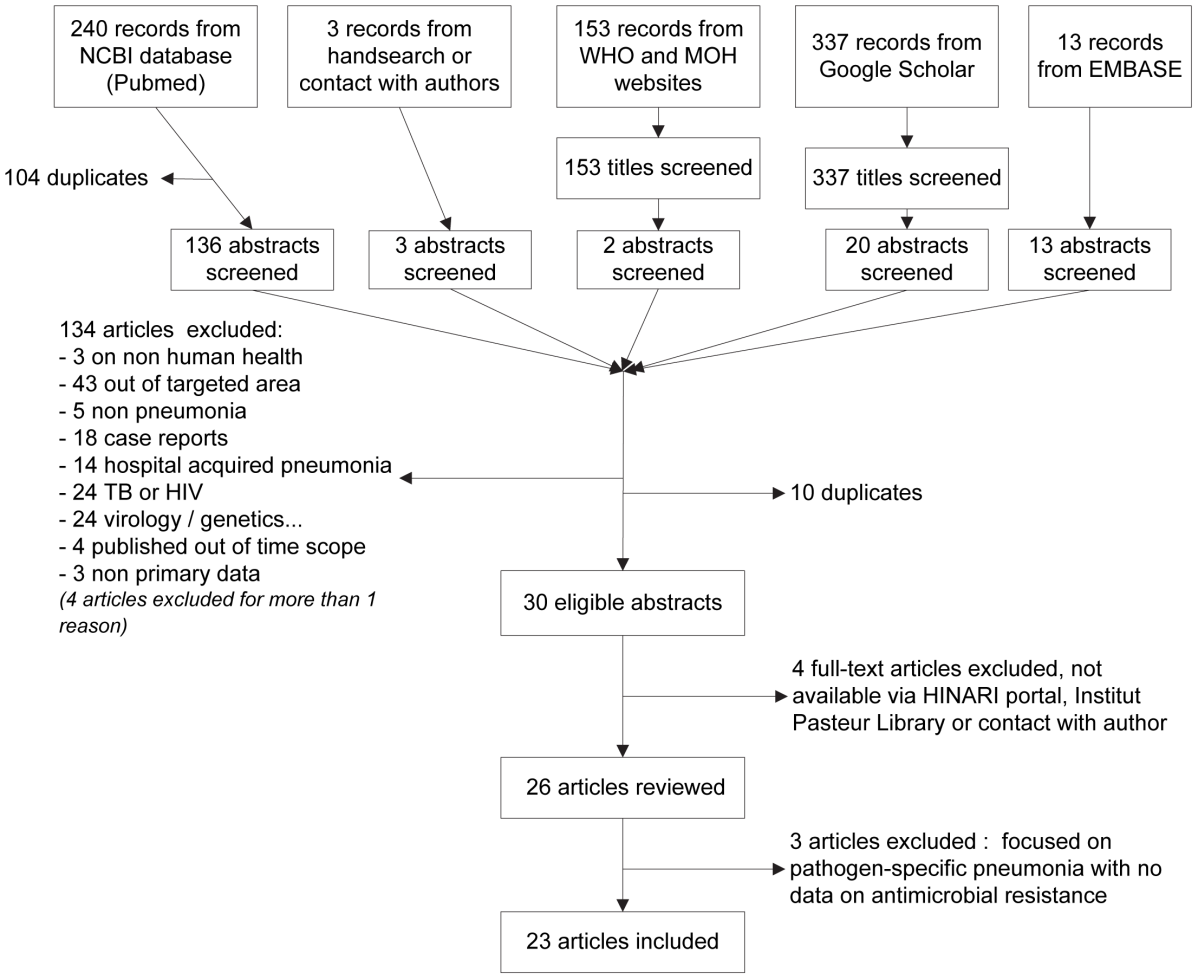
Inclusion flow chart.

The initial search in NCBI, Google Scholar, WHO and MOH and EMBASE databases and the hand search initially retrieved 240, 337, 153, 13 and 3 references, respectively (Figure 2). We removed duplicates and publications not fitting with our inclusion criteria and obtained 30 eligible papers, of which 26 could be accessed and analyzed. From these, three articles were excluded as they were focused on a pathogen-specific pneumonia and did not report antibiotic resistance [29], [30], [31]. Ultimately, 23 articles were included. From 15 of them, we extracted information on CAP etiologies [32], [33], [34], [35], [36], [37], [38], [39], [40], [41], [42], [43], [44], [45], [46]. Eight presented antibiotic resistance data [18], [46], [47], [48], [49], [50], [51], [52]. We did not find published articles describing CAP in Laos.

### 2. “Grey Data” Inclusions

**Figure 3:**
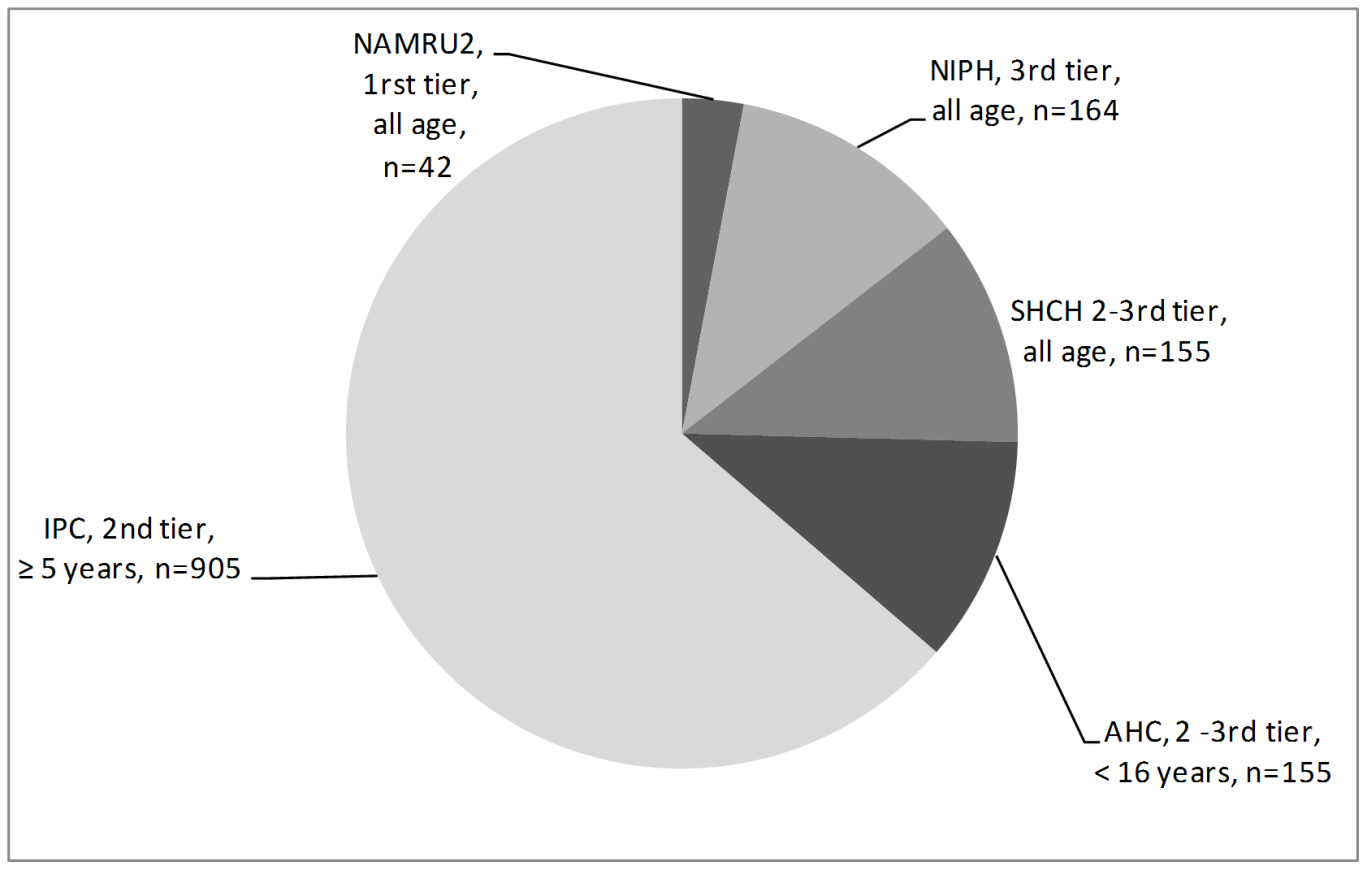
Sources of unpublished data from Cambodia and number of patients with bacterial etiologies explored.

Five datasets were included (Figure 1). AHC reported information on 155 bacteremic children admitted with signs of LRI or presenting respiratory complications of an underlying infectious disease [22]. IPC provided information for 1,904 acute LRIs in patients aged ≥5 years, including 959 (50.4%) CAP cases. Bacterial etiologies were explored for 905 (94.4%) of these [23]. NAMRU2 shared laboratory results on 42 febrile patients with cultures positive for potential respiratory pathogens. The SHCH blood stream infection (BSI) surveillance reported 155 adult patients with confirmed BSI and LRI clinical signs. The NIPH surveillance contributed data from 164 severe LRI patients (Figure 3).

### 3. Study Population

The studies presenting etiologies included a total of 5919 pediatric or adult patients. Of these, 4498 (76%) were enrolled from May 1995 to April 2008 in Thailand [33], [34], [35], [36], [37], [38], [39], [40], [41], [42], [46] and Vietnam [32], [43], [44],[45], and 1421 were patients treated in Cambodia between January 2007 and December 2012 (Table S1).

Five sources reported on patients of all age (NAMRU2, NIPH and three published papers [36], [37], [38]). Nine sources reported on adults or children >5 years (SHCH, IPC, [33], [35], [39], [40], [41], [43], [46], three on children aged <15 (AHC, [34], [42]) and three on children aged <5 [32], [44], [45].

Fourteen studies related to inpatients only (AHC, IPC, NIPH, [32], [33], [35], [36], [37], [38], [40], [41], [42], [43], [44]) two on outpatients only (NAMRU2, [39]), and three on both (SHCH, [34], [46]).

### 4. Studies’ Characteristics

The median duration for all studies considered was 24 months (Interquartile range: 18–36 months). The published studies were surveillance studies (n = 10), cross sectional (n = 3) and case-control studies (n = 2).

The risk of bias for estimating bacterial etiologies of CAP was considered high in 5/15 published studies presenting etiologies (mostly due to lack of details in the descriptions of study participants’ selection procedures or of laboratory techniques) and in 1/5 of unpublished sources of data in Cambodia (because of a broad case definition) (Table S2).

CAP etiologies were determined by culture, antigen and serological tests, and molecular diagnostic tools (Table S1). In Cambodia, bacterial pathogens were identified using culture: on blood samples (all except NAMRU-2) and on sputum and/or pleural fluid samples (all except AHC and SHCH). In Thailand and Vietnam, cultures were performed on blood, sputum and pleural fluid samples in nine, seven, and four out of the 15 published studies respectively. Serology tests using agglutination methods were performed on paired sera (n = 9), urine (n = 4) or pleural fluid (n = 1). Antigen tests were performed on urine samples (n = 3), on serum (n = 1) or pleural fluid (n = 1). In 6 studies, polymerase chain reaction (PCR) and Real Time-PCR were performed on naso-pharyngeal swabs or aspirates. Of the 13 studies conducted in Thailand and Vietnam, four sought to identify atypical pathogens only.

### 5. Bacterial Etiologies

The White literature showed that *Streptococcus pneumoniae* (*S. pneumoniae* ) and *Haemophilus influenzae* (*H. influenzae)* were among the 5 most prevalent bacteria in, respectively, seven and six of the nine published studies using regular cultures to determine bacterial CAP etiologies (Table S1).

In 3^rd^-tier hospitals, *S. pneumoniae* ranked first or second in the list of the most prevalent bacteria in all CAP studies. *S. pneumoniae* was cultured from sputum or blood samples of 29.1% of adults with bacteremic pneumonia in one study [43]. In 2011, in a 3^rd^-tier Vietnamese study, *S. pneumoniae* was found in 38.7% of naso-pharyngeal swabs of children with pneumonia [45]. *H. influenzae* was also prevalent in all 3^rd^-tier settings, the highest rate was 31.8% in adult outpatients cases in Thailand (2002–3) with fever and signs of pneumonia [39], [41]. *H. influenzae* was also discovered using PCR in 50.0% of the naso-pharyngeal swabs collected in Vietnamese children with pneumonia [45]. *Klebsiella pneumoniae (K. pneumoniae)* was among the most common pneumonia pathogens in four of the eight articles reporting data on 3^rd^-tier level., all of these studied pneumonia in adults [39], [40], [41], [43]Other bacteria described were *B. pseudomallei*, *H. parainfluenzae* and atypical pathogens such as *Mycoplasma pneumoniae* (*M. pneumoniae*), *Chlamydia pneumoniae* (*C. pneumoniae)*, *Moraxella catarrhalis* [32], [34], [39], [40], [41], [42], [43], [45].

In 2^nd^-tier CAP inpatients, Gram-negative bacteria were also commonly isolated. *K. pneumoniae* was found among the five most frequent bacteria in all three articles describing etiologies of radiologically-confirmed CAP, using cultures as diagnostic tools. Its prevalence reached 35.5% in positive sputum cultures in one setting in Thailand [35]. *B. pseudomallei* was also among the most frequent pathogens, found in up to 24.0% of blood cultures of bacteremic patients in one setting [38] and in 7.7% of positive sputum cultures in another setting [35]. *S. pneumoniae* was identified in 22.4% of admitted patients with CAP in one setting [46]and 17% of bacteremic patients in another [38]. Other causes included *H. influenzae, Staphylococcus aureus, Escherichia coli, Acinetobacter* spp. and *Pseudomonas aeruginosa* [33]. Studies looking for atypical pneumonia pathogens at the 2^nd^-tier level identified *Legionella longbeachae*, *M. pneumoniae*, *C. pneumoniae*, *Coxiella burnetti* and *Legionella pneumophilla* [36], [37], [46].

Grey data gathered in Cambodia showed that 30.1% of all-age febrile patients seen at primary healthcare level with a positive sputum culture were infected by *S. pneumoniae* (NAMRU2) (Table S1). At the 2^nd^-tier level of care, in two provincial hospitals, the most prevalent bacteria cultured from blood and sputum samples of patients with radiologically confirmed CAP were *H. influenzae*, *K. pneumoniae*, *B. pseudomallei*, *S. pneumoniae* and *P. aeruginosa* (cultured in 5.7%, 3.1%, 2.8%, 2.4% and 2.1% of their samples, respectively) (IPC). In 3^rd^-tier care level hospitals, *S. pneumoniae* was the most commonly encountered pneumonia pathogen. It was the most frequent pathogen in children admitted to one hospital (AHC) and the third most frequent in another surveillance (NIPH). *S. pneumoniae* was, however, not among the most prevalent pathogens in the 3^rd^-tier setting (SHCH); though in this setting, a quarter of BSI-confirmed patients had taken antibiotics prior to admission [53]. Other frequent bacteria included *S. aureus* (AHC, SHCH), *E. coli* (NIPH, SHCH), *K. pneumoniae* (AHC, NIPH) and *B. pseudomallei* (AHC, NIPH, SHCH). Finally, other Gram-negative bacteria were isolated in small numbers: *Acinetobacter baumanii* and *Salmonella enteric* Serotype Choleraesuis.

### 6. Antimicrobial Resistance

Antimicrobial resistance of *S. pneumoniae*, *H. influenzae, K. pneumoniae* and *B. pseudomallei* are presented in Tables 1, 2, 3 and 4, respectively. Tests and interpretative breakpoints used are reported in the tables’ footnotes where available.

**Table 1:** Antimicrobial resistance rates of *S. pneumonia.*

Antimicrobial agent	Resistance rate a : %										Mean resistance rate b			
study reference	[41]	[50]	[46]	[48]	[43]	[39]	NAMRU	IPC	SHCH	AHC				
study period	′99–′00	′95–′04	′01–′02	′02–′03	′02–′04	′03–′04	07–′10	07–′10	07–′10	07–′11				
n. of isolates	30	64	53	200	57	12	16	84	4	26	n		N	%
penicillin G														
intermediate resistance			37.7	29.0	17.5			51.0	1/4	0.0	132	/	424	31.1
high level resistance		34.4	26.4	31.0	35.1			4.0	1/4	19.0	123	/	488	25.2
resistance (level not defined)	13.3					16.6	0.0				6	/	58	58.0
ampicillin		1.6						11.0			10	/	148	6.9
amoxicillin								6.0			5	/	84	6.0
amoxicillin-clavulanic acid				0.0	14.0						8	/	257	3.1
cefuroxime				47.5	47.4						122	/	257	47.5
ceftriaxone			5.7		33.3			0.0	0.0	0.0	22	/	224	9.8
cephalothin		0.0									0	/	64	0.0
cefotaxime				24.5				10.0			57	/	284	20.2
chloramphenicol		12.5		78.5			12.0	13.0		40.0	188	/	390	48.3
tetracycline		26.6					81.0	33.0			58	/	164	35.2
erythromycine		20.6	100.0	55.5			47.0	47.0	1/4	32.0	233	/	447	52.1
azithromycin				49.5							99	/	200	49.5
vancomycin								2.0			2	/	84	2.0
thrimetoprim/sulfamethoxazole		51.6	100.0	76.5			62.0	92.0	3/4		329	/	421	78.2
ofloxacin				37.5							75	/	200	37.5
levofloxacine				1.0			0.0				2	/	216	0.9
lincomycin		18.7								0.0	12	/	90	13.3
clindamycin				32.0			31.0				69	/	216	31.9

Notes:

a Number of resistant strains/total number of strains in each study, expressed as %. Methods and breakpoints applied:

[41] Methods and breakpoints: not defined.

[50] Methods: disk diffusion and Etest (penicillin): breakpoint penicillin intermediate resistance MIC 0.12-1 µg/ml; resistance ≥2.0 µg/ml (NCCLS/CLSI 2002 M100 S12).

[46] Methods: agar dilution; breakpoint penicillin intermediate resistance MIC 0.12-1 µg/ml, resistance ≥2.0 µg/ml (NCCLS/CLSI 2001M7-A4).

[48] Methods: disk diffusion and Etest; breakpoints penicillin intermediate resistance: MIC 0.12-1 µg/ml, resistance ≥2 µg/ml (CLSI 2006 M100-S16).

[43] Methods: broth microdilution; breakpoints penicillin intermediate resistance: 0.12-1 µg/ml; resistance: MIC ≥2 µg/ml (CLSI).

[39] Method: disk diffusion; breakpoints according to NCCLS/CLSI (details not given).

NAMRU: Methods: disk diffusion and Etest; breakpoints (CLSI 2007).

IPC: Methods: disk diffusion and Etest; breakpoint penicillin intermediate resistance: MIC >0.06 mg/l, resistance >2 mg/l (Recommendations Société Française de Microbiologie (RSFM) 2007).

SH AHC: Methods: disk diffusion and Etests; breakpoints (CLSI 2011).

ACH: Methods: disk diffusion and Etest; breakpoints (CLSI 2012).

b Sum of all resistant organisms/sum of all organisms tested.

**Table 2:** Antimicrobial resistance rates of *H. influenza.*

Antimicrobial agent	Resistance rate a : %						Mean resistance rate b			
study reference	[41]	[43]	[39]	NAMRU	IPC	AHC				
study period	′99–′00	′02–′04	′03–′04	′07–′12	′07–′10	′07–′11				
n. of isolates	15	16	14	14	167	14	n		N	%
ampicillin	33.0			57.1	42.0	61.0	92	/	210	43.6
amoxicillin					13.0		22	/	167	13.0
amoxicillin-clavulanic acid		0.0		21.4	2.0	0.0	6	/	211	3.0
imipenem		0.0		0.0	1.0		2	/	197	0.8
meropenem				14.3			2	/	14	14.3
azithromycin		12.5					2	/	30	6.7
cefotaxime					0.0		0	/	167	0.0
cefuroxime	0.0	6.3		14.3			3	/	45	6.7
ceftriaxone		6.3				0.0	0	/	30	0.0
ceftazidime				14.3			2	/	14	14.3
cephalotin					36.0		60	/	167	36.0
cefepime				7.1			1	/	14	7.1
gentamicin					3.0	50.0	12	/	181	6.6
chloramphenicol	0.0			42.8	46.0	67.0	92	/	210	43.9
tetracycline				93.0	83.0		152	/	181	83.8
erythromycin	33.4		57.0				13	/	29	44.8
thrimetoprim/sulfamethoxazole	47.0		64.0	42.8	83.0	78.0	172	/	224	76.8
clarithromycin	0.0						0	/	15	0.0
ciprofloxacin		0.0		0.0			0	/	30	0.0
levofloxacin				0.0			0	/	14	0.0

Notes:

a Number of resistant strains/total number of strains in each study, expressed as %.

[41] Methods and breakpoints: details not given.

[43] Methods: broth microdilution; (CLSI)[39] Method: disk diffusion; breakpoints according to NCCLS/CLSI (details not given).

NAMRU: Methods: disk diffusion and Etest; breakpoints (CLSI 2007).

IPC: Methods: disk diffusion and Etest; breakpoints (RSFM 2007).

SHCH: Methods: disk diffusion and Etest; breakpoints (CLSI 2012).

AHC: Methods: disk diffusion and Etests; breakpoints (CLSI 2011).

b Sum of all resistant organisms/sum of all organisms tested.

**Table 3:** Antimicrobial resistance rates of *K. pneumonia.*

Antimicrobial agent	Resistance rate a : %					Mean resistance rate b			
study reference	[41]	[43]	[18]	SHCH	AHC				
study period	′99–′00	′02 –′04	′07–′10	07–′10	07–′11				
n. of isolates	25	36	47	6	9	n		N	%
amoxicillin				100.0		6	/	6	100.0
amoxicillin-clavulanic acid		22.2	21.3	33.3	66.0	26	/	98	26.5
imipenem		0.0	2.1		0.0	1	/	92	1.1
meropenem				0.0		0	/	6	0.0
cefalothin	4.0					1	/	25	4.0
cefuroxime		11.1				4	/	36	11.1
cefotaxim			19.2 (n = 38 tested)		100.0	18	/	56	32.2
ceftriaxone		2.8		66.7	75.0	12	/	51	23.1
ceftazidime			19.1		100.0	18	/	56	32.1
cefepime			23.6			11	/	47	23.6
cefpodoxime					86.0	8	/	9	86.0
gentamicin	0.0		17.0	16.7	63.0	15	/	87	16.9
amikacin	0.0		2.1	0.0		1	/	78	1.3
chloramphenicol					67.0	6	/	9	67.0
colistin					0.0	0	/	9	0.0
thrimetoprim/sulfamethoxazole			45.7 (n = 35 tested)	83.3	63.0	32	/	62	51.9
ciprofloxacin		0.0	18.6	16.7	50.0	14	/	98	14.5

Notes:

a Number of resistant strains/total number of strains in each study, expressed as %.

[41] Methods and breakpoints: details not given.

[43] Methods: broth microdilution; (CLSI).

[18] Methods: disk diffusion and Etest; breakpoints (RSFM 2007).

SHCH: Methods: disk diffusion and Etest; breakpoints (CLSI 2012).

AHC: Methods: disk diffusion and Etests; breakpoints (CLSI 2011).

b Sum of all resistant organisms/sum of all organisms tested.

**Table 4:** Antimicrobial resistance rates of *B. pseudomallei.*

Antimicrobial agent	Resistance rate a : %					Mean resistance rate b			
study reference	[41]	[52]	[47]	[51]	AHC				
study period	′99–′00	′96–′02	′07–′10	′07–′10	07–′11				
n. of isolates	20	125	54	39	6	n		N	%
amoxicillin-clavulanic acid			0.0	18.0	0.0	7	/	99	7.1
imipenem				0.0	0.0	0	/	45	0.0
meropenem			0.0			0	/	54	0.0
thrimetoprim/sulfamethoxazole			0.0	0.0	33.0	2	/	99	2.0
ceftazidime	0.0	0.0	0.0	0.0	0.0	0	/	244	0.0
cefoperazone-sulbactam		0.0				0	/	125	0.0
tetracycline				3.0		1	/	39	3.0
doxycycline			0.0		0.0	0	/	60	0.0
cotrimoxazole	0.0	0.0	0	0.0	33.0	2	/	244	0.8
chloramphenicol	0.0	0.0	22.2	3.0	0.0	13	/	244	5.4

Notes:

a Number of resistant strains/total number of strains in each study, expressed as %.

[41] Methods and breakpoints: details not given.

[52] Methods and breakpoints: details not given.

[47] Methods: disk diffusion and Etest; breakpoints (CLSI 2012).

[51] Methods: disk diffusion and Etest; breakpoints (RSFM 2007).

AHC: Methods: disk diffusion and Etests; breakpoints (CLSI 2011).

b Sum of all resistant organisms/sum of all organisms tested.

*S. pneumoniae* displayed little resistance to penicillin A (mean 6.4%, range 1.6–11.0), to amoxicillin-clavulanic acid (mean 3.1%, range 0.0–14.0), various degrees of resistance to cephalosporins (to ceftriaxone: mean 9.8%, range 5.7–33.3, to cefuroxime: mean 47.5%, range 47.5–47.4), moderate resistance to chloramphenicol (mean 48.3%, range 12.0–78.5) and high level of resistance to trimethoprim/sulfamethoxazole (SXT) (mean 78.2%, range 51.6–100.0).

*H. influenzae* displayed high levels of resistance to SXT in our results (mean76.8%, range 42.8–83.0), with a rising trend over time. Overall susceptibility to amoxicillin, to amoxicillin-clavulanic acid and to cephalosporins was preserved.

Of all 98 *K. pneumoniae* isolates tested in Thailand, Vietnam or Cambodia, 26.5% showed resistance to amoxicillin-clavulanic. Resistance of *K. pneumoniae* to cephalosporins was reported but carbapenems and amino-glycosides remained generally active on most isolates. Ureidopenicillin and carboxypenicillin were unfortunately not tested.

Finally, some *B. pseudomallei* isolates did not show susceptibility to amoxicillin-clavulanic acid and chloramphenicol and are naturally resistant to all aminoglycosides and macrolides, but preserved susceptibility to ceftazidime, carbapenems and SXT, which are the main therapeutic agents.

## Discussion

CALIBAN findings indicate that pathogens isolated in patients with CAP in Cambodia do not seem to differ from those described in published studies from neighboring or Western countries [54].

### 1. Pathogens

*S. pneumoniae* and *H. influenzae* were found to be the two most common bacterial etiologies of pneumonia in Cambodia (aside from *M. tuberculosis*) and in the lower Mekong region. This is in line with the global findings that *S. pneumoniae* causes about half of the severe forms of CAP in children <5 years worldwide, while 20% may be due to *H. influenzae* type b. These pathogens account for 13.8 million cases and 7.9 million cases of disease respectively globally in children under five years of age in 2010 [55], [56]. It is estimated that they are responsible for 2.3 and 1.4 million all age deaths respectively annually in the Western Pacific WHO Region alone [55], [56].

Irrespective of patients’ age, Gram-negative bacteria such as *K. pneumoniae* and *B. pseudomallei* were also frequently found particularly in bacteremic cases [18], [30], [47], [51]. *B. pseudomallei* was the only pathogen causing CAP in the region which does not widely circulate in developed countries [39], [41], [52], [57], [58]. Melioidosis has recently been described as a frequent, endemic pathogen in Cambodia, but its true incidence in the community remains unknown [30], [47], [51], [59].

The epidemiology of atypical CAP in Cambodia remains unclear due to the current lack of data. However, this information is pivotal to guide clinical practice and antimicrobial choices, as atypical pneumonia has sometimes been associated with severe clinical presentations in Thailand [37], [42]. The required techniques for atypical CAP direct diagnosis are costly and not routinely available. Studies relying on indirect laboratory diagnostic methods showed that *M. pneumoniae* and *C. pneumoniae* were the most prevalent atypical pneumonia pathogens, with *C. pneumoniae* most frequent in adults [43], while *M. pneumoniae* was more common in children and adolescents [42].

### 2. Antimicrobial Resistance

It is clear that *S. pneumoniae* is the most frequently identified pathogen causing CAP and that high-level penicillin resistance may not be as high as presumed [60]. Therefore data from Cambodia, Thailand and Vietnam suggest that amoxicillin probably remains a valid option for empirical treatment of CAP. Instead, *S. pneumoniae* displayed high levels (∼75%) of resistance to trimethoprim/sulfamethoxazole in all studies from Cambodia, Thailand and Vietnam included in this review precluding its empirical use for CAP. A close surveillance of resistance patterns in *S. pneumoniae* is highly warranted.

*H. influenzae* and *B. pseudomallei* strains isolated in most hospitals remained sensitive to amoxicillin-clavulanic acid, but severe forms of *B. pseudomallei* pneumonia require other agents *i.e.* ceftazidime or carbapenems.

Multidrug resistant bacteria producing carbapenemases such as New Delhi metallo-beta-lactamase-1 (NDM-1) have been reported in Asian countries in every clinical setting, including CAP [61]. In China, 2% of 208 *K. pneumoniae* isolates from 12 hospitals produced carbapenemases [62]. Carbapenemase-producing *K. pneumoniae* isolates from various samples were also detected in South Korea, in Singapore and in Taiwan [63], [64], [65]. In Thailand NDM-1-producing *K. pneumoniae* isolates were reported from urinary samples [66]. Our review did not show any report of carbapenemase-producing enterobacteriaceae to December 2012 in Cambodia and neighboring countries in respiratory samples. Only strains of *K. pneumoniae* resistant to amoxicillin-clavulanic acid and to third-generation cephalosporins were responsible for CAP. The emergence of multidrug resistant enterobacteriaceae should be closely monitored.

### 3. Empirical Therapy

Publications on antimicrobial resistance (AMR) in Asia and elsewhere do describe alarming levels of resistance in various pathogens [67], [68]. They are, however, often based on data from reference laboratories or hospitals which sample patients – who are often hospitalized and have advanced disease. This major surveillance bias may incite clinicians and policymakers to resort to broad spectrum antibiotics such as fluoroquinolones as first-line treatment. Our data on CAP in the community suggest that fluoroquinolones are not warranted as a first-line treatment in the majority of cases. These antibiotics are very effective but they are expensive, have side effects and may be a powerful tool against tuberculosis in highly endemic settings such as Cambodia [69], [70], [71]. Preserving fluoroquinolones from emerging resistance is therefore a priority to help in the fight against severe bacterial infection and multidrug resistant tuberculosis [72]. Prevention of AMR development can only be attained through continued clinical evaluation of patients during the first 48 hours, and through improved antibiotic stewardship in the healthcare setting – both public and private - as well as improved regulation of unprescribed, over-the-counter sales. Empiric treatment schedules should also take into account the presence of difficult-to-treat Gram-negative bacteria such as *B. pseudomallei* and *K. pneumoniae*, particularly in diabetics (particularly for *B. pseudomallei*) and in the elderly (*K. pneumoniae*). Easy-to-use diagnostic tests to identify patients with multi-drug resistant bacteria requiring broad spectrum antibiotics are needed, despite the challenges associated with their development.

### 4. Limitations

Our review has certain limitations due to the patchy availability of data (Table S1). Most sources of data have focused on CAP inpatients, biasing findings toward severe presentations. Our review analyzed few data on children at 2^nd^-tier hospital level, which probably represents a high caseload in Cambodia. It also lacked data on atypical pathogens and data on viral-bacterial co-infections.

Our review of CAP etiologies was further limited by the various diagnostic methods used. Most studies relied on blood cultures which have a high specificity but low sensitivity and may over-represent severe patients with CAP caused by pyogenic bacteria [73]. Moreover, the causal role of cultured bacteria in pneumonia, though highly likely, may be arguable in some cases, especially in the elderly with co-morbidities. The usefulness of sputum cultures to identify pneumonia etiologies has been debated, particularly in children <5 years old as they may not discriminate between infection and throat colonization [73], [74].

Some of the studies we reviewed used indirect laboratory techniques, such as serological tests. Authors working on blood sera collected paired samples to measure the rise in antibodies but did not systematically report the exact timing of the convalescent sample although it influences the sensitivity and specificity of this test [75]. Moreover, these techniques tend to be positive during colonization so these results may be less reliable than cultures [76].

Despite these limitations, the value of this review is to sketch out the epidemiological situation which may be useful for prescription in the daily clinical setting. This should also inspire future studies and surveillance. In particular the potential development of high-level penicillin resistance in *S. pneumoniae* and the potential spread of complex resistance mechanisms in Gram-negative pathogens (*e.g. ESBL* and carbapenemases) should be followed closely.

### 5. Perspective

#### From a clinical standpoint

Based on these findings and on the experience of the CALIBAN clinicians, amoxicillin remains indicated in the first line treatment options for CAP in Cambodia, unless anamnestic and clinic findings raise the suspicion of CAP caused by *B. pseudomallei* or ESBL-producing strains of *K. pneumoniae*. Well-designed studies can be implemented in hospitals participating in this network to define the patients most at risk for such difficult-to-treat CAP.

Pneumonia remains a major public health issue despite efforts undertaken within the scope of the Millennium Development Goals [1]. The basic epidemiological information needed to plan effective health interventions is lacking most in less developed countries [77], in which guidelines from Western countries cannot simply be transposed. Our project aimed to fill this important knowledge gap in Cambodia.

#### From an epidemiological and public health standpoint

The data from Cambodia was examined in light of regional data. Many of these studies were conducted in Thailand and Vietnam some years ago. It is somewhat reassuring that the current Cambodian situation appears similar to that described in these countries several years earlier. The epidemiology of CAP may have evolved somewhat since that time in the countries described. Regularly reviewing data from those countries might provide insights into the road lying ahead for Cambodia, where surveillance systems must be set up to monitor AMR in the community.

Many developing countries have a trove of data which is not identified, structured and analyzed in a systematic way to inform policymakers. In the absence of well-conducted, clinically and biologically well-documented prospective studies, a systematic review of available national data complemented with published regional epidemiological information helps clinicians make informed choices. Our findings on CAP etiologies and resistance profiles were shared with the Cambodian health authorities to guide the revision of national empiric treatment and were uploaded on a specially created website for easier access and ability to download [78].

In the Cambodian context without surveillance of bacterial pathogens and resistance patterns at national level, this approach of systematically reviewing grey and published data, linking epidemiologists and clinicians to contextualize and gain a fair view of the CAP situation was useful, rapid (a few months) and cost-effective. Finally, it laid the foundations for future collaboration within CALIBAN, a new network on antimicrobial resistance and pulmonary infection.

## Supporting Information

Table S1 **Most frequent pathogens isolated in patients with CAP, in Cambodia and neighboring countries, a review of published and unpublished data.** Notes: #: *L. pneumophila* and *L. longbeachae* were only tested on 554 severe CAP. $ : including 48 patients HIV+ and 15 diabetics. a: *S. pneumoniae*+*H. influenza.* c: *H. influenzae*+*M. pneumonia.*
(XLSX)

Table S2 **Risk of bias.** Notes: **Risk of bias: High indicates bias in each domain. Moderate suggests potential bias in each domain. Low excludes bias in each domain. # Summary of risk of bias: High risk ≥1 high risk of potential bias; or >2 moderate risks of potential bias. Moderate risk >1 moderate risk of potential bias. Low risk: Low risk in all criteria, or <2 moderate risk.
(XLSX)

Checklist S1 **Prisma check list.**
(DOCX)

Document S1 **Pubmed search on bacterial lung infections in the Mekong Region, performed in May 2012.**
(DOCX)
