# Supplementary material for: Etiologies and Resistance Profiles of Bacterial Community-Acquired Pneumonia in Cambodian and Neighboring Countries’ Health Care Settings: A Systematic Review (1995 to 2012)
Source: PLoS One. 2014 Mar 13;9(3):e89637. doi: 10.1371/journal.pone.0089637 (PMC3953073; doi:10.1371/journal.pone.0089637)
Supplement: Document S1 — Pubmed search on bacterial lung infections in the Mekong Region, performed in May 2012. (DOCX) [file pone.0089637.s004.docx]

**Supplemental document S1: Pubmed search on bacterial lung infections in the Mekong Region, performed in May 2012**

**Search 1**

Search: ((“Pneumonia”[Mesh] OR “Pneumonia, Bacterial”[Mesh] AND “Pneumonia, Pneumococcal”[Mesh] OR “microbiology”[Subheading])) AND (“Mekong Valley”[Mesh] OR “Asia, Southeastern”[Mesh])

Search through all NCBI databases

>> 22 hits in Pubmed, no other sources in other databases

**Search 2**

Same on forgotten AND changed to OR

Search: ((“Pneumonia”[Mesh] OR “Pneumonia, Bacterial”[Mesh] OR “Pneumonia, Pneumococcal”[Mesh] OR “microbiology”[Subheading])) AND (“Mekong Valley”[Mesh] OR “Asia, Southeastern”[Mesh])

>> 136 hits including 91 duplicates references

**Search 3**

Same but Vietnam, Thailand or Cambodia instead of Mekong

Search: ((“Pneumonia”[Mesh] OR “Pneumonia, Bacterial”[Mesh] OR “Pneumonia, Pneumococcal”[Mesh] OR “microbiology”[Subheading])) AND (“Cambodia”[Mesh] OR “Vietnam”[Mesh] OR “Thailand”[Mesh] OR “Laos”[Mesh])

>> 82 hist including 13 duplicates references

**Additional search performed in January 2013, in EMBASE database**

Database(s): Embase Classic+Embase 1947 to 2014 Week 01

**Search Strategy**

| **#** | **Searches** | **Results** |
| --- | --- | --- |
| 1 | community acquired pneumonia/dr, ep, et [Drug Resistance, Epidemiology, Etiology] | 1950 |
| 2 | Cambodia/ | 2816 |
| 3 | Laos/ | 1349 |
| 4 | Viet Nam/ | 10621 |
| 5 | Thailand/ | 23080 |
| 6 | 1 and 2 | 0 |
| 7 | 1 and 3 | 0 |
| 8 | 1 and 4 | 1 |
| 9 | 1 and 5 | 12 |
| 10 | 6 or 7 or 8 or 9 | 13 |
